# Supplementary material for: MixTrain: accelerating DNN training via input mixing
Source: Front Artif Intell. 2024 Sep 4;7:1387936. doi: 10.3389/frai.2024.1387936 (PMC11443600; doi:10.3389/frai.2024.1387936)
Supplement: Supplementary file 1 [file Data_Sheet_1.pdf]

MixTrain: Accelerating DNN Training via Input Mixing

Correspondence*:

1 APPENDIX

1.1 Experimental Setup

This subsection describes the experimental setup used for realizing the baseline and proposed training schemes, on the benchmarks specified in Section of the main paper. We conduct our experiments on the complete training and test datasets of each benchmark, using the PyTorch (Paszke et al., 2019) framework.

Baseline: We consider SGD training as the baseline in our experiments. The hyper-parameters used in SGD training of each of the benchmarks are described below.

ImageNet: For experiments in Section 5.1 we utilize a batch-size of 64 per GPU, for all benchmarks. For the ResNet18, ResNet34 and ResNet50 benchmarks the initial learning rate set to 0.025. The learning rate is decreased by 0.1 every 30 epochs, for a total training duration of 90 epochs, and the weight decay is $4e-5$. The MobileNetV2 benchmark utilizes an initial learning rate of 0.0125. We use a cosine learning rate decay schedule, as in (Li et al., 2019) for 150 epochs. The weight decay is set to $4e-5$. All benchmarks use an input size of 224*224*3.

Cifar10-CNNs: All experiments on the convolutional neural networks (i.e., ResNet18 and ResNet34) utilize a batch-size of 128, trained on a single GPU. We consider two different hyper-parameter settings that differ in the learning rate schedule used. When using a linear learning rate schedule, all benchmarks are trained with an initial learning rate of 0.05 that is decayed by 0.1 every 10 epochs, across 90 epochs. The cosine annealing learning rate schedule uses an initial learning rate of 0.1, that is gradually decayed over 200 epochs. Across all experiments, the weight decay is set to $5e-4$. All benchmarks utilize an input size of 32*32*3.

Cifar10-Transformers: We consider three vision transformer architectures, ViT-small, ViT-SWIN and ViT-pretrained. The ViT-small architecture has a patch-size of (4*4), with the hidden dimension size equal to 512. The network consists of 8 attention heads, and a depth of 6. The ViT-SWIN architecture is identical to the Swin-T architecture in (Liu et al., 2021). When training from scratch, both networks operate on inputs of size (32*32*3), and are trained for 100 epochs using a cosine annealing learning rate schedule, with the initial learning rate of 1e-4. For the fine-tuning experiment, the ViT-pretrained network uses the ViT-B/16 architecture described in (Dosovitskiy et al., 2020). Here the pretrained weights are obtained by training on the ImageNet-21k dataset (Deng et al., 2009), and the network hence accepts an input of size (384*384*3). Fine-tuning is conducted for 3 epochs. For all models, training is conducted across 4 GPUs, with the batch-size set to 128.

mixTrain: mixTrain uses the same learning rate, weight decay, and number of epochs as baseline SGD, requiring no additional hyper-parameters. We use the same random seed for both our baseline and mixTrain experiments. Results in Sec. 5.1 are reported by averaging over 3 different training runs.

Table 1. Training CNNs on Cifar10 using mixTrain

Network	Training Strategy	Top-1 Error	Speed-Up
ResNet18	Baseline SGD (linear learning rate schedule)	6.5%	1×
	mixTrain-`CutMix`	**5.4%**	**1.74**×
	mixTrain-`MixUp`	**5.7%**	**1.69**×
ResNet18	Baseline SGD (cosine annealing learning rate schedule)	4.4%	1×
	mixTrain-`CutMix`	**4.2%**	**1.45**×
	mixTrain-`MixUp`	**4.33%**	**1.41**×
ResNet34	Baseline SGD (linear learning rate schedule)	5.2%	1×
	mixTrain-`CutMix`	**4.2%**	**1.78**×
	mixTrain-`MixUp`	**4.6%**	**1.71**×

Table 2. mixTrain with different optimizers

Network	Training Strategy	Top-1 Error	Speed-Up
ResNet18	Baseline Adam	6%	1×
	mixTrain-`CutMix`	**5.8%**	**1.59**×
	mixTrain-`MixUp`	**5.92%**	**1.51**×
ResNet18	Baseline AvaGrad	5.7%	1×
	mixTrain-`CutMix`	**5.2%**	**1.42**×
	mixTrain-`MixUp`	**5.4%**	**1.4**×
ResNet18	Baseline AvaGrad-W	5.8%	1×
	mixTrain-`CutMix`	**5.28%**	**1.44**×
	mixTrain-`MixUp`	**5.3%**	**1.39**×

1.2 Experimental Results on Cifar10

To underscore the wide applicability of mixTrain, we present our runtime and accuracy trade-off achieved on the Cifar10 benchmarks in Table 1. Across our benchmarks, `MixUp` achieves upto 1.7 × improvement in runtime, while `CutMix` achieves a 1.8× runtime improvement. Clearly, both mixing strategies provide a boost in accuracy, due to the improved regularization provided via mixing samples.

As can be seen in Table 2, we also highlight the applicability of mixTrain to optimizers such as Adam (Kingma and Ba, 2017) and AvaGrad (Savarese et al., 2019), and different learning rate schedules (Loshchilov and Hutter, 2016). Typically, such optimizers propose techniques to evaluate the weight gradients in a manner that results in faster convergence. MixTrain does not interfere with the evaluation of such weight gradients- regardless of the optimizer used, MixTrain achieves training acceleration by reducing the effective size of the dataset to iterate over each epoch. As can be seen, MixTrain can be successfully applied in conjunction with such optimizers.

1.3 Analysis of Top-5 accuracy without interference reduction

In Sec. 4.1 we mention that the network appears to be unable to detect both constituent inputs when interference is not reduced. At most, the network detects only one of the constituent inputs, with the second constituent rarely appearing in the Top-5 predictions made. We provide the Top-5 classification accuracy of the second constituent, prior to reducing interference.

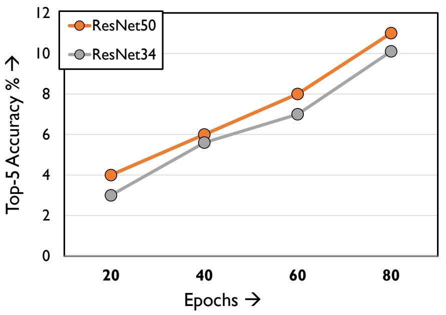

Figure 1. Top-5 classification accuracy of second constituent input

This necessitates the need for devising strategies to reduce interference between the constituent inputs of a composite sample.

1.4 Training runtime

We present our training runtime results in Table 3. Note that the Cifar10 experiments are conducted on a single Nvidia RTX 2080Ti machine, while the ImageNet experiments are conducted across 4 RTX gpus.

Table 3. Training runtime

Network	Training Strategy	Runtime
ResNet18-Cifar10	Baseline-SGD	3.6 hours
	mixTrain-**CutMix**	1.95 hours
ResNet18-ImageNet	Baseline-SGD	30 hours
	mixTrain-**CutMix**	20.1 hours
ResNet50-ImageNet	Baseline-SGD	51 hours
	mixTrain-**CutMix**	32.7 hours

1.5 Analyzing mixTrain when mixing more than 2 inputs

mixTrain can be extended to beyond 2 samples. However, we observe that the effect of mixing more than two samples is different for different benchmarks. Table 4 below shows the performance of mixTrain when 2 and 3 samples are mixed using the Cut-Mix operator, on the Cifar10 and ImageNet datasets for the ResNet18 network. For the Cifar10-ResNet18 benchmark, mixing N=3 samples clearly provides better runtime savings than N=2, and at comparable accuracy to baseline. However, we observe a noticeable drop in accuracy for the ImageNet benchmark. In the context of Cut-Mix, this is because the class object of interest occupies a smaller fraction of the input area for ImageNet, and is likely to be missed in the random Cut-Mix patch. We note that we observe similar trends in accuracy when using the Mix-Up operator. Here, the interference between constituent inputs is higher due to averaging pixel information across more samples.

1.6 Analysis of loss across consecutive epochs

As mentioned in Sec. 4.2, we utilize the loss of a sample in epoch E to determine it's amenability to mixing in epoch E+1. However, several mini-batches pass before a sample is trained again in the next epoch. As the model undergoes many changes to its weights, it is possible that the loss of a sample in epoch E might be quite substantially different from that in epoch E+1.

Table 4. Mixing more than 2 inputs using mixTrain

Network	Training Strategy	Top-1 Acc	Speed-Up
ResNet18-Cifar10	Baseline SGD (N=0)	95.6%	1×
	mixTrain-**CutMix** (N=2)	**95.8%**	**1.45**×
	mixTrain-**CutMix** (N=3)	**94.4%**	**2.32**×
ResNet18-ImageNet	Baseline SGD (N=0)	69.8%	1×
	mixTrain-**CutMix** (N=2)	**69.56%**	**1.5**×
	mixTrain-**CutMix** (N=3)	**68.7%**	**1.9**×

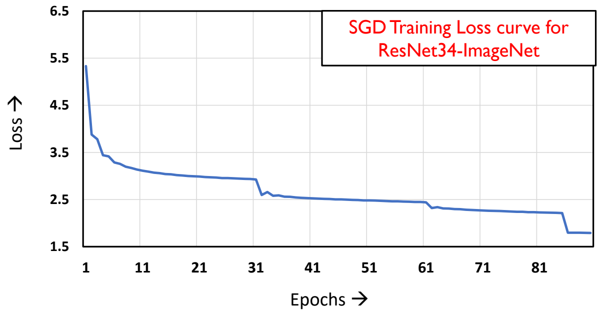

Figure 2. Change in average loss across epochs

Fig. 2 plots the loss curve averaged across all training examples when trained with SGD. The loss appears to change rapidly only for the first few epochs, and later when the learning rate changes. In other periods, changes in loss happen more gradually. We find that the same analysis is generally applicable when samples are mixed as well. This thus justifies using the loss in epoch E to justify amenability in epoch E+1.

1.7 Analyzing efficacy of amenability metric

As part of our key strategies to achieve a good accuracy versus runtime efficiency trade-off, we propose selectively mixing samples in Section 4.2. We take into consideration the region of the loss distribution where the sample occurs in epoch E to appropriately decide whether the sample should be mixed in the next epoch. Each region differs based on the estimated impact mixing a sample may have on accuracy. Consequently, each region has its own criteria for gauging amenability for the next epoch.

In Table 5, we compare our proposed selective mixing strategy against the following set of rules to gauge amenability.

Table 5. Analyzing efficacy of our amenability metric

Benchmark	Amenability Metric	Top-1 err	Speed-Up
ResNet50	Our Effort (Region 1 only)	24.56%	1.38
	Our Effort (Regions 1 and 3)	24.45%	1.56
	Threshold = Accuracy	24.80%	1.36
	Threshold = Average Loss	24.50%	1.33
	Region 1 and threshold = L_{incorr} for Region 3	25.14%	1.74

- First, we analyze the trade-off achieved when we mix inputs that were correctly classified in previous epoch, instead of using L_{mid} as in Sec. 4.2. Essentially, only those samples that are correct in epoch E are mixed in the next epoch. We find that the L_{mid} threshold (Row 1) achieves slightly better classification accuracy, as outlier inputs with correct classification are avoided.
- Next, we compare against an average loss threshold, i.e., we calculate the running average of the loss across all the samples in S_{noMix}. If a sample in epoch E has loss lower than the running average, it is mixed in the next epoch and vice-versa. As can be seen, our L_{mid} threshold (Row 1) achieves better speed-ups for nearly the same classification accuracy. Across epochs, classification accuracy improves and average loss reduces, often with several correctly classified samples with loss above the average loss. This metric thus loses the opportunity to approximate training effort on these these correctly classified samples that are amenable to interpolation.
- Finally, we compare the efficacy of our Region 3 criterion. We observe the trade-off achieved when all samples above L_{incorr} are mixed, in addition to Region 1 approximations. Clearly, our proposed criterion attains better accuracy (Row 2).

1.8 Discussion on applicability of mixTrain to other domains

We now demonstrate the applicability of mixTrain to other domains such as natural language processing (NLP).

Interpolation-based data augmentation techniques have been successfully applied when training NLP tasks such as test classification (Wang et al., 2018). The different interpolation techniques proposed vary in the manner in which the inputs are mixed. For example, (Guo et al., 2019; Sawhney et al., 2022) demonstrate that interpolation can be performed both in the input word embedding space, as well as by using the feature outputs of a hidden layer. In the first approach, (word mix-up), random input sentence pairs are selected for mixing during each mini-batch iteration, similar to (Zhang et al., 2017). Each sentence in the selected pair is padded to the same length, and words at the same index across each sentence are linearly averaged in some ratio. In contrast, the second approach, i.e., sentence mix-up, performs interpolation by linearly averaging the features of the selected sentence pairs. Typically, the features provided as input to the softmax layer are chosen for mixing.

For better training runtime efficiency during mixTrain, it is crucial that the inputs are processed in a mixed form across a majority of the network layers. We thus demonstrate mixTrain by linearly averaging the word embeddings, as in word mix-up. Table 6 highlights the runtime efficiency of mixTrain across the CoLA and SST-2 datasets from the GLUE benchmark (Wang et al., 2018). All training hyper-parameters are set to that in (Sawhney et al., 2022), and no additional hyper-parameter tuning is required. As can be seen, mixTrain has negligible impact on accuracy, thereby underscoring the widespread applicability of the proposed approach.

Table 6. Analyzing efficacy of mixTrain on NLP tasks

Network (Dataset)	Training Technique	F1-score	Speed-Up
	Baseline	84.41	1
Bert-base (CoLA)	MixTrain-MixUp	84.12	1.26
	Baseline	90.12	1
Bert-base (SST-2)	MixTrain-MixUp	90.01	1.2

REFERENCES

Deng, J., Dong, W., Socher, R., Li, L.-J., Li, K., and Fei-Fei, L. (2009). ImageNet: A Large-Scale Hierarchical Image Database. In *CVPR09*

KEY:
ANNOTATION:

Dosovitskiy, A., Beyer, L., Kolesnikov, A., Weissenborn, D., Zhai, X., Unterthiner, T., et al. (2020). An image is worth 16x16 words: Transformers for image recognition at scale. *CoRR* abs/2010.11929

KEY:
ANNOTATION:

Guo, H., Mao, Y., and Zhang, R. (2019). Augmenting data with mixup for sentence classification: An empirical study. *CoRR* abs/1905.08941

KEY:
ANNOTATION:

[Dataset] Kingma, D. P. and Ba, J. (2017). Adam: A method for stochastic optimization

KEY:
ANNOTATION:

Li, D., Zhou, A., and Yao, A. (2019). Hbonet: Harmonious bottleneck on two orthogonal dimensions. In *The IEEE International Conference on Computer Vision (ICCV)*

KEY:
ANNOTATION:

Liu, Z., Lin, Y., Cao, Y., Hu, H., Wei, Y., Zhang, Z., et al. (2021). Swin transformer: Hierarchical vision transformer using shifted windows. *CoRR* abs/2103.14030

KEY:
ANNOTATION:

Loshchilov, I. and Hutter, F. (2016). SGDR: stochastic gradient descent with restarts. *CoRR* abs/1608.03983

KEY:
ANNOTATION:

Paszke, A., Gross, S., Massa, F., Lerer, A., Bradbury, J., Chanan, G., et al. (2019). Pytorch: An imperative style, high-performance deep learning library. In *Advances in Neural Information Processing Systems 32*, eds. H. Wallach, H. Larochelle, A. Beygelzimer, F. d Alché-Buc, E. Fox, and R. Garnett (Curran Associates, Inc.). 8024–8035

KEY:
ANNOTATION:

Savarese, P., McAllester, D., Babu, S., and Maire, M. (2019). Domain-independent dominance of adaptive methods. *CoRR* abs/1912.01823

KEY:
ANNOTATION:

Sawhney, R., Thakkar, M., Pandit, S., Soun, R., Jin, D., Yang, D., et al. (2022). DMix: Adaptive distance-aware interpolative mixup. In *Proceedings of the 60th Annual Meeting of the Association for Computational Linguistics (Volume 2: Short Papers)* (Dublin, Ireland: Association for Computational Linguistics), 606–612. doi:10.18653/v1/2022.acl-short.67

KEY:
ANNOTATION:

Wang, A., Singh, A., Michael, J., Hill, F., Levy, O., and Bowman, S. R. (2018). GLUE: A multi-task benchmark and analysis platform for natural language understanding. ArXiv preprint 1804.07461

KEY:
ANNOTATION:

Zhang, H., Cissé, M., Dauphin, Y. N., and Lopez-Paz, D. (2017). mixup: Beyond empirical risk minimization. *CoRR* abs/1710.09412

KEY:
ANNOTATION:
